# Increased EGFR/HER2 Pathway Activation Contributes to Skin Tumorigenesis in *Tpl2*^−^^/−^ Mice

**DOI:** 10.3390/cancers17203362

**Published:** 2025-10-18

**Authors:** Laura R. Purkey, Stefania Mehedincu, Charles Irvine, Raelyn Akdag, Megan Little, W. Wade Kothmann, Katharine Rus, Erin Greenberg, Neil Shady, Kathleen DeCicco-Skinner

**Affiliations:** 1 Department of Biology, American University, Washington, DC 20016, USAci8670a@american.edu (C.I.); ra5576a@american.edu (R.A.);; 2 Department of Biochemistry and Molecular & Cellular Biology, Georgetown University, Washington, DC 20057, USA

**Keywords:** *Tpl2*, miR-205, cSCC, EGFR, ErbB2, HER2, MAP3K8, miR-21

## Abstract

**Background:** The mitogen-activated protein kinase (MAPK) signaling pathway is frequently dysregulated in cutaneous squamous cell carcinoma (cSCC). Tumor progression locus 2 (*Tpl2*), a serine/threonine protein kinase within the MAPK family, regulates cellular proliferation, survival, and inflammatory responses. Loss of *Tpl2* activates compensatory signaling cascades, driving increased papilloma and cSCC development. In this study we examined whether dysregulated ErbB signaling contributes to the enhanced tumor burden found in *Tpl2*^−/−^ mice. **Methods**: To evaluate whether aberrant ErbB signaling drives tumorigenesis in *Tpl2*^−/−^ mice, wild-type (*Tpl2*^+/+^) and Tpl2^−/−^ mice were subjected to a two-stage chemical carcinogenesis protocol for 48 weeks. A subset of mice received Gefitinib (an EGFR inhibitor) or Lapatinib (a HER2 inhibitor) in their diet. **Results**: We found that *Tpl2* ablation increases gene expression of EGFR, HER2, and HER3, while baseline protein levels remain unchanged between *Tpl2* genotypes. To investigate the possibility of microRNA (miR)-mediated post-transcriptional regulation of EGFR, HER2, and HER3, we measured ErbB-related miR expression in keratinocytes. We found that HER2/3-related miRs 205 and 21 are increased in *Tpl2*^−/−^ keratinocytes. Further, *Tpl2* loss enhances p-EGFR, EGFR, and HER2 protein expression in papillomas. and HER2-related microRNAs (miRs) 205 and 21 in keratinocytes, and enhances p-EGFR, EGFR, and HER2 protein expression in papillomas. *Tpl2*^−/−^ mice developed 12-fold more papillomas and 4-fold more cSCCs compared to *Tpl2*^+/+^ animals. Treatment with Gefitinib or Lapatinib reduced papilloma numbers by 88% and 50%, respectively, while restoring cSCC numbers to *Tpl2*^+/+^ levels. **Conclusions**: These findings indicate that ErbB targeting represents a promising therapeutic strategy for cSCCs arising from MAPK pathway dysregulation.

## Simple Summary

The mitogen-activated protein kinase (MAPK) signaling pathway plays a critical role in cellular growth and survival, and its disruption is associated with various cancers. Loss or alteration of critical regulatory proteins in this pathway can trigger compensatory mechanisms that activate alternative signaling routes that promote tumor development. This study demonstrates that loss of a specific MAPK family member, *Tpl2*, leads to upregulation of growth factor receptors and associated signaling molecules, resulting in dramatically increased tumor formation in mice. Importantly, targeted inhibition of these upregulated receptors significantly reduced tumor burden, suggesting that therapeutic strategies focused on blocking compensatory signaling pathways could be valuable for treating cancers that arise from MAPK pathway dysfunction.

## 1. Introduction

Cutaneous squamous cell carcinoma (cSCC) is the second most prevalent form of skin cancer globally [1,2]. There has been a 300% increase in cSCC diagnoses over the past three decades, and this upward trajectory is projected to continue, driven by factors such as heightened exposure to ultraviolet and chemical carcinogens, as well as an aging population [2,3]. While surgery is effective for most patients with cSCC, those with metastatic and advanced cases have few treatment options with poor outcomes [4]. Further, a high mutation rate complicates the identification of key driver mutations [5].

The mitogen-activated protein kinase (MAPK) pathways, which are frequently dysregulated in cSCC, serve as essential regulators of numerous cellular processes, including proliferation, survival, differentiation, and inflammatory responses [6]. These sophisticated, interconnected networks respond to various stimuli, such as growth factors, stress signals, and inflammatory mediators [7]. Upon a ligand binding to its receptor, RAS is activated and triggers a phosphorylation cascade in which MAP3K phosphorylates MAP2K, and subsequently phosphorylates and activates MAPK. Once activated, MAPK translocates to the nucleus to regulate target gene expression.

Tumor progression locus 2 (*Tpl2*) is a gene encoding a serine/threonine kinase that plays an important role in inflammation, immunity, and cancer [8,9]. As a MAP3K, *Tpl2* lies upstream of both the MAP2K MEK1/MEK2 and the MAPK ERK1/ERK2. Our prior research demonstrated that *Tpl2* ablation in mice increases sensitivity to aberrant RAS signaling, resulting in heightened inflammation and tumorigenesis [10]. Additionally, the absence of *Tpl2* leads to increased activation of several receptor-tyrosine kinase (RTK) bypass pathways, including those associated with the Epidermal Growth Factor Receptor (EGFR/ErbB) family [11,12]. The extent to which dysregulated EGFR signaling is responsible for the heightened tumorigenesis in *Tpl2*^−/−^ mice is unknown.

The ErbB family consists of four transmembrane receptors: ErbB1 (EGFR/HER1), ErbB2 (HER2), ErbB3 (HER3), and ErbB4 (HER4) [13]. These RTKs function through ligand-induced homo- or heterodimerization, leading to activation of downstream signaling pathways that promote cell proliferation, survival, and migration. Overexpression of EGFR in high-risk or metastatic cutaneous squamous cell carcinoma (cSCC) is associated with poor prognosis, and emerging evidence suggests that EGFR inhibitors may offer potential benefit for patients with advanced disease [14,15,16]. Likewise, HER2 mutation or amplification is reported in non-melanoma skin cancers and HER2 loss significantly reduces DMBA/TPA-induced skin tumor formation [17,18].

MicroRNAs (miRNAs or MiRs) are short (~22 nucleotide) non-coding RNAs that are frequently dysregulated in cancer [19]. By binding to target mRNAs, microRNAs can suppress gene expression at the post-transcriptional level. In the skin, they serve as regulators in processes such as wound healing, epidermal differentiation, and the response to ultraviolet radiation [15]. Notably, they demonstrate dual functionality in cSCC, with some functioning as oncogenic miRNA (OncomiR) while others act as tumor suppressors [20]. Several microRNAs, including miR-7, miR-125, miR-21, and miR-205, regulate ErbB signaling by targeting expression of the receptors themselves or downstream components of their pathways [20,21].

In this study, we found heightened gene expression of ErbB receptors and ErbB-related microRNA 205 and 21 in keratinocytes from *Tpl2*^−/−^ mice. Additionally, immunohistochemical analysis of papillomas from *Tpl2*^−/−^ mice showed upregulation in EGFR, p-EGFR and HER2. Using a DMBA/TPA multi-stage chemical carcinogenesis protocol, we found that *Tpl2*^−/−^ mice developed significantly more tumors and experienced more rapid tumorigenesis compared to control mice. Mice fed the EGFR inhibitor Gefitinib reduced tumor burden by 88% in *Tpl2*^−/−^ mice, while the HER2 inhibitor Lapatinib achieved a 50% tumor reduction. The findings indicate that *Tpl2* loss removes safeguards that normally limit ErbB-driven oncogenic processes, establishing the ErbB family as a key mechanistic link between *Tpl2* deficiency and accelerated carcinogenesis.

## 2. Materials and Methods

### 2.1. In Vivo Tumor Experiment

Sixty male and female wild-type (*Tpl2*^+/+^) and knockout (*Tpl2*^−/−^) C57Bl/6 mice engineered as previously described were maintained at the American University (Washington, DC, USA) animal facility [22]. For two-stage chemical carcinogenesis studies, mice were randomized into groups (8–10 mice/group) matched for age, weight, and sex. Prior to initiation with 7,12-dimethylbenz(a)anthracene (DMBA; 100 μg/200 μL acetone), dorsal skin was shaved. Mice were promoted with twice weekly application of TPA painted on the skin (10 μg/200 μL acetone) for 20 weeks. At the time of promotion, mice were fed ad libitum an AIN-93G diet or AIN-93G diet containing 200 mg/kg Gefitinib, an EGFR tyrosine kinase inhibitor, or 200 mg/kg Lapatinib, an EGFR/HER2-targeted tyrosine kinase inhibitor. Control mice treated with only acetone, DMBA, or TPA were maintained for both genotypes. Mice were monitored daily for general appearance and tumor measurements and weights taken weekly. Tumor-bearing animals were individually housed to avoid injury to the tumor sites. Animals were euthanized 48 weeks after the date of initiation, or at an earlier time point if the animal was deemed moribund. At the study endpoint, portions of skin and tumors were either snap-frozen for DNA/RNA/protein isolation or formalin-fixed for Immunohistochemistry. Tumors underwent a histological examination in a blinded fashion by a certified pathologist to determine tumor type (Applied Pathology Systems; Shrewsbury, MA, USA).

### 2.2. Primary Keratinocyte Isolation and Treatment

Primary keratinocytes were isolated from *Tpl2*^−/−^ and wild-type *Tpl2*^+/+^ C57Bl/6 mice pups between 1 and 4 days old as previously described [23]. Keratinocytes were grown at 37 °C and 5% CO_2_ in keratinocyte growth media (Promocell; Heidelberg, Germany) containing hormone supplements, Penicillin-Streptomycin (10,000 U/mL) and low (0.06 mM) calcium. For in vitro drug studies, keratinocytes were treated with 1 μM Lapatinib or 1 μM Gefitinib (Selleck Chemicals; Houston, TX, USA) for 15 min (for assessing phosphorylated protein) or 24 h (for assessing total protein) prior to isolation.

### 2.3. RT-qPCR

RNA was isolated using Invitrogen’s PureLink RNA Mini Kit (Thermofisher Scientific; Waltham, MA, USA) and quantified with a NanoVue Plus Spectrophotometer (GE Healthcare (Chicago, IL, USA)). For ErbB receptor expression, cDNA was reverse transcribed from template RNA and qPCR was performed as previously described using specific forward and reverse primers for each gene target, with GAPDH as the housekeeping gene (Table 1) [24]. For microRNA gene expression, RNA was standardized to 100–125 ng/mL before reverse transcription using the Qiagen miRCURY LNA RT kit (Qiagen; Germantown, MD, USA). qPCR was performed with Qiagen’s miRCURY LNA SYBR Green PCR kit using miR-21a-3p (YP00205400) and miR-205-3p (YP02114511) sequences (Table 2). MiRNA abundance was determined by absolute quantification using synthetic oligonucleotides with serial dilutions of 10 mM to 0.001 mM (miR205) and 1 mM to 0.0001 mM (miR21). Pooled keratinocyte RNA from 4 to 6 mice was used, with experiments repeated a minimum of three times.

### 2.4. Immunohistochemistry

Immunohistochemistry was conducted as previously described [25]. Sections of skin, tumors, and SCCs from *Tpl2*^−/−^ and *Tpl2*^+/+^ mice, fixed in formalin and embedded in paraffin, were sectioned into 4 mm slices and stained with hematoxylin and eosin (H&E). Primary antibodies targeting the ErbB receptors along with anti-rabbit secondary antibodies (Cell Signaling #8339) were applied and used at concentrations of 1:200 to 1:600. Sections came from a minimum of three individual mice per treatment group. Representative areas were captured using 10× and 50× magnification.

High-magnification images were analyzed using the Fiji distribution of ImageJ2 (Version 2.17.0). Regions of interest (ROIs) encompassing areas of dense chromogen deposition were identified using empirically determined thresholds applied to pixel intensity, and then the total area within all ROIs was summed for each image. Identical thresholds were used for all images collected from papilloma and cSCC samples. Due to the weak immunolabeling of samples from normal skin, a different threshold was applied to these images in an attempt to minimize the inclusion of unlabeled cells that strongly took up the H&E stain; even with a more stringent threshold, the quantification values likely overrepresent the total area of chromogen deposition in the normal skin samples. Some images contained dark areas that were clearly not part of the tissue section (e.g., cellular debris from processing) or the structure of interest (e.g., a hair bulb near a papilloma or cSCC), but which registered as an ROI due to their average pixel intensity. When these areas were 100% identifiable and separable from the actual ROIs labeled with chromogen, they were manually cropped from the image before analysis. Quantification of IHC images is shown in Supplementary Materials.

### 2.5. Western Blotting

Keratinocyte protein was extracted using RIPA lysis buffer with protease/phosphatase inhibitors and quantified by BCA assay (Thermo Fisher; Waltham, MA, USA). 25 μg protein was electrophoresed using 4–12% Tris-Glycine gels, transferred to PVDF membranes, and blocked with 5% milk/BSA. Primary antibodies (EGFR, HER2, HER3, p-EGFR, β-actin, GAPDH; Cell Signaling Technologies, Danvers, MA, USA) were used at 1:500-1:1000, and secondary anti-rabbit HRP was applied at 1:2000. Bands were detected with West Dura substrate (Thermo Fisher), quantified using NIH ImageJ, and normalized to β-actin/GAPDH. Pooled protein from 4 to 6 mice was used with experiments repeated a minimum of 3 times.

### 2.6. Statistical Analysis

Data was tested for normality, model assumptions were checked, and the data were analyzed with SPSS software, version 28.01.01. For qPCR data, unpaired *t*-tests were used for statistical analysis. Tumor induction experiments were analyzed through two-way ANOVA with Tukey’s post hoc test. Significance for all analyses was assumed at a *p*-value of 0.05 or less. Significance values of *p* ≤ 0.05 are indicated in figures with a single asterisk (*), *p* ≤ 0.01 with a double asterisk (**), and *p* ≤ 0.001 with a triple asterisk (***).

## 3. Results

### 3.1. ErbB Receptor Upregulation in Tpl2^−/−^ Keratinocytes and Compensatory Response to Inhibitor Treatment

**Figure 1:**
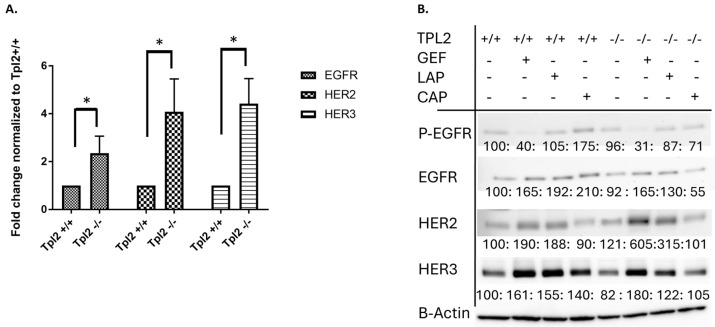
ErbB Receptor Upregulation in *Tpl2*^−/−^ Keratinocytes and Compensatory Response to Inhibitor Treatment. (**A**) Real-time PCR analysis of EGFR, HER2, and HER3 expression in primary keratinocytes from *Tpl2*^+/+^ and *Tpl2*^−/−^ keratinocytes. Error bars represent SD. (**B**) Western analyses for p-EGFR, EGFR, HER2 and HER3. *Tpl2*^+/+^ and *Tpl2*^−/−^ keratinocytes were cultured in low calcium growth media and upon 90% confluency treated with vehicle-control, 1 μM Lapatinib or 1 μM Gefitinib for 15 min (for phosphorylated protein) or 24 h (for assessing total protein) prior to isolation. NIH Image J was used for densitometry and bands were normalized to beta-actin, which served as a houskeeping gene. Densitometry changes in normalized protein (% of *Tpl2*^+/+^ control) are displayed below each blot. Original western blots are presented in Supplementary Materials.

To assess whether the ErbB receptor tyrosine kinases EGFR, HER2, and HER3 were upregulated in *Tpl2*^−/−^ keratinocytes, qPCR was performed (Table 1, Figure 1). *Tpl2*^−/−^ keratinocytes exhibited a 2.4-fold increase in EGFR mRNA expression compared to *Tpl2*^+/+^ keratinocytes (Figure 1A). Similarly, *Tpl2*^−/−^ keratinocytes exhibited a 4.1-fold higher expression of HER2 mRNA and 4.4-fold higher expression of HER3 mRNA than *Tpl2*^+/+^ keratinocytes. Despite upregulation in gene expression, baseline protein levels in *Tpl2*^+/+^ and *Tpl2*^−/−^ keratinocytes were similar, suggesting post-transcriptional modulation (Figure 1B). Inhibition of p-EGFR using Gefitinib led to compensatory upregulation of EGFR family receptors in both *Tpl2*^+/+^ and *Tpl2*^−/−^ keratinocytes. In *Tpl2*^+/+^ cells, Gefitinib treatment increased EGFR, HER2, and HER3 protein expression by 1.7-, 1.9-, and 1.6-fold, respectively. *Tpl2*^−/−^ cells showed a similar pattern but with more pronounced HER2 upregulation (1.7-, 6.1-, and 1.8-fold increases for EGFR, HER2, and HER3, respectively). Lapatinib treatment produced comparable compensatory effects. *Tpl2*^+/+^ keratinocytes treated with Lapatinib demonstrated 1.9-, 1.9-, and 1.6-fold increases in EGFR, HER2, and HER3 protein, respectively, while *Tpl2*^−/−^ cells showed increases of 1.3-, 3.2-, and 1.2-fold, respectively. Given our previous findings that MET receptor (another RTK) is also activated in *Tpl2*^−/−^ keratinocytes, we also tested whether Capmatinib (a MET inhibitor) could increase ErbB receptors [11]. Capmatinib treatment increased p-EGFR, EGFR, and HER3 protein levels in *Tpl2*^+/+^ mice (1.7-,2.1-, and 1.4-fold, respectively), but not in *Tpl2*^−/−^ keratinocytes (Figure 1B).

### 3.2. miR205 and miR21 Are Upregulated in Tpl2^−/−^ Keratinocytes

**Figure 2:**
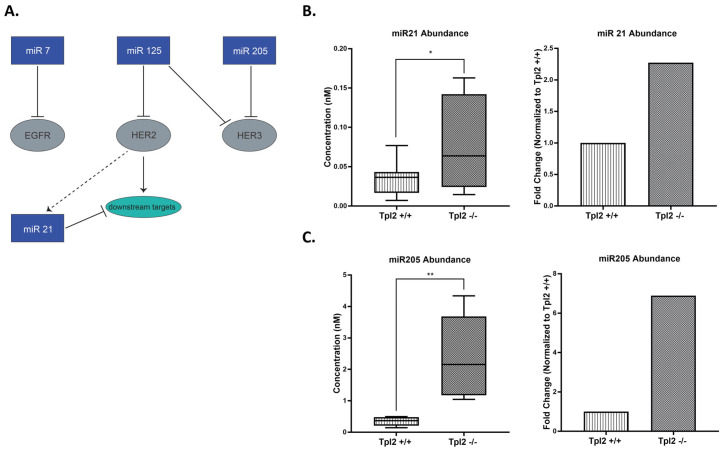
ErbB receptors (EGFR, HER2, HER3) and microRNAs 205 and 21 are upregulated in keratinocytes from *Tpl2*^−/−^ mice. (**A**) Schematic of ErbB-related miRs 7, 125, 21, and 205 and their targets. Dashed line denotes that HER2 overexpression can modulate levels of miR21 (**B**) abundance (in nanomolar concentration) of miR21 and respective fold change (**C**) abundance (in nanomolar concentration) of miR205 in *Tpl2*^+/+^ and *Tpl2*^−/−^ keratinocytes and respective fold change. Box and whisker plots show the median miR abundance, with box edges representing the 25th and 75th quartiles. Respective fold change displays mean miR abundance.

Given their established role in post-transcriptional regulation, microRNA analysis was performed to explore potential mechanisms underlying the observed gene-protein expression differences. Several miRNAs—namely miR-205, miR-7, miR-21, and miR-125—have been identified as key modulators of ErbB family members and play critical regulatory roles in cSCC (Figure 2A). Thus, we tested their expression in *Tpl2*^+/+^ and *Tpl2*^−/−^ keratinocytes. qPCR analysis revealed significant microRNA upregulation in *Tpl2*^−/−^ keratinocytes: miR-21 was increased 2.3-fold (*p* = 0.049) and miR-205 was increased 6.9-fold (*p* = 0.003) compared to *Tpl2*^+/+^ keratinocytes (Figure 2B,C). These results were consistent across a minimum of four independent biological replicates. miR-125 showed no genotype differences and miR-7 was barely detectable.

### 3.3. Pharmacological Inhibition of EGFR or HER2 Decreases Tumorigenesis in Tpl2^−/−^ Mice

**Figure 3:**
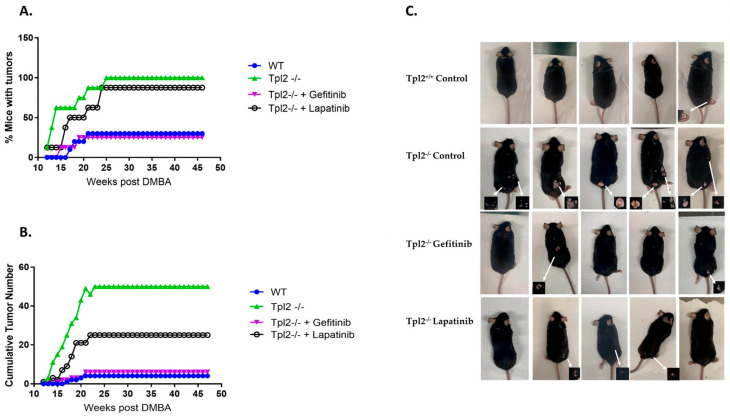
Pharmacological inhibition of EGFR or HER2 decrease skin tumor formation in *Tpl2*^−/−^ mice. Mice were treated once with 100 μg DMBA/200 μL acetone. Promotion was induced one week later by application of 10 μg TPA/200 μL acetone and continued twice/week for 20 weeks. At the time of promotion mice (*n* = 8–10/treatment) were fed ad libitum AIN-93G diet or AIN-93G diet containing 200 mg/kg Gefitinib, an EGFR tyrosine kinase inhibitor, or 200 mg/kg Lapmatinib, an EGFR/HER2 targeted tyrosine kinase inhibitor. (**A**) The percentage of tumor-bearing mice and (**B**) the number of tumors per mouse are plotted vs. time for each group. (**C**) Representative photograph of five of the DMBA/TPA mice per group.

To investigate the role of EGFR/HER2 signaling in tumorigenesis, we used a two-stage chemical carcinogenesis model in *Tpl2*^+/+^ and *Tpl2*^−/−^ mice. Like our prior work, 100% of *Tpl2*^−/−^ mice developed skin tumors compared to only 30% of wildtype (Tpl2^+/+^) mice (Figure 3A). Tumor latency was significantly shorter in *Tpl2*^−/−^ mice (*p* < 0.0001), with 63% developing papillomas by week 14 compared to 0% of *Tpl2*^+/+^ mice. Tumor burden was also dramatically higher in *Tpl2*^−/−^ mice (54 tumors total) compared to *Tpl2*^+/+^ mice (4 tumors total) (*p* < 0.001; Figure 3B). Gefitinib and Lapatinib treatment reduced tumor burden in *Tpl2*^−/−^ mice by 88% and 50%, respectively, restoring tumor counts to *Tpl2*^+/+^ levels (Figure 3B,C). Histological analysis revealed a higher incidence of cSCCs in the Tpl2^−/−^ mice, with 4 observed compared to only 1 in the *Tpl2*^+/+^ mice. Notably, treatment with either Gefitinib or Lapatinib reduced the number of cSCCs in the *Tpl2*^−/−^ mice to 1 in each treatment group (Table 3).

### 3.4. Papillomas from Tpl2^−/−^ Mice Have Increased Expression of EGFR, p-EGFR and HER2

**Figure 4:**
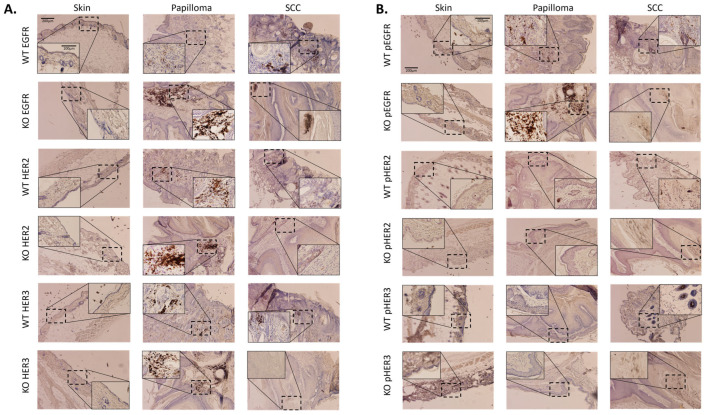
Papillomas and cSCCs from *Tpl2*^−/−^ mice have increased expression of p-EGFR, EGFR, HER2, and HER3. (**A**,**B**) Skin, papillomas, and cSCCs were obtained from *Tpl2*^+/+^ and *Tpl2*^−/−^ mice and probed for p-EGFR, EGFR, p-HER2, HER2, p-HER3, and HER3 using Immunohistochemistry. Images at 10× and 50× are displayed. A scale bar is depicted in the WT (*Tpl2*^+/+^) skin images. For IHC analysis, a minimum of three tissue sections from three individual mice were examined. Scale bars = 200 μm (main panels) and 100 μm (insets).

Skin, papillomas, and cSCCs were obtained from *Tpl2*^+/+^ and *Tpl2*^−/−^ mice and were stained for EGFR, p-EGFR, HER2, p-HER2, HER3 or p-HER3. *Tpl2*^−/−^ papillomas showed increased expression of EGFR, p-EGFR, and HER2 compared to *Tpl2*^+/+^ mice (Figure 4A,B). cSCCs had lower expression of all ErbB-related receptors compared to papillomas, and similar expression patterns between genotypes, suggesting that upregulation of RTKs in Tpl2^−/−^ papillomas may be required for malignant transformation to cSCCs. For IHC analysis, a minimum of three tissue sections from three individual mice were examined. Since only one cSCC developed in *Tpl2*^+/+^ mice in this study, two additional sections from *Tpl2*^+/+^ cSCCs obtained from a parallel study were included to ensure reproducibility.

## 4. Discussion

Growing evidence suggests that cSCC development and progression may arise from aberrant MAPK signaling pathways. [5,26] While MAPK inhibitors show promise as a cancer therapeutic strategy, their clinical application is complicated by the development of secondary malignancies [7,27]. This paradox is particularly evident in BRAF-mutant melanoma, where these inhibitors effectively target critical oncogenic pathways yet simultaneously increase the risk of cSCC [27]. This effect is also evident following *Tpl2* (MAP3K) loss, as we have previously reported that *Tpl2* ablation results in a significantly higher number of papillomas and cSCCs [10,11].

Here we demonstrate that MAP3K signal disruption can upregulate compensatory ErbB pathways that maintain pro-tumorigenic signaling. Tpl2 ablation leads to increased gene expression of EGFR, HER2, and HER3 in Tpl2^−/−^ keratinocytes and increased EGFR, p-EGFR, and HER2 protein levels in *Tpl2*^−/−^ papillomas. EGFR signaling drives proliferation and pro-inflammatory cytokine/chemokine upregulation, promoting tumorigenesis and therapy resistance [13]. This is consistent with our previous findings of increased tumorigenesis and inflammation in *Tpl2*^−/−^ mice [10,11,25].

Upon administration of Gefitinib or Lapatinib to keratinocytes, HER2 and HER3 signaling pathways become upregulated, indicating the activation of compensatory bypass mechanisms. This adaptive response demonstrates the tumor’s ability to circumvent targeted therapeutic blockades by redirecting cellular signaling through alternative receptor networks. While this occurred in both genotypes, the upregulation in HER2 signaling following Gefitinib or Lapatinib treatment was more pronounced in *Tpl2*^−/−^ keratinocytes.

MicroRNAs are key regulators in cSCC, particularly through their influence on ErbB signaling. Among the miRNAs implicated, miR-205, miR-7, miR-21, and miR-125 play prominent roles in modulating ErbB receptor activity [20]. miR-7 and miR-125 typically function as tumor suppressors by targeting EGFR or HER2/3, respectively, or their downstream signaling components [28]. In contrast, miR-21 is frequently overexpressed in cSCC [20,29,30]. miR-205 demonstrates context-specific behavior, acting as either a tumor suppressor or promoter depending on the tissue type. [20] However, in cSCC, MiR-205 is oncogenic, as it is reported to be more frequently expressed in cSCCs with aggressive traits, including association with local recurrence, promotion of keratinocyte migration, and correlation with clinical indicators of poor patient outcomes [29,31,32]. Consistent with these findings, we observed significantly elevated levels of miR-205 and miR-21 in keratinocytes derived from Tpl2^−/−^ mice. Although we observed upregulation of two miRNAs, we acknowledge this does not provide direct functional evidence of their ability to inhibit ErbB signaling in our model. Future studies will employ miRNA overexpression/knockdown approaches and investigate additional post-transcriptional mechanisms to establish clearer causal relationships and determine which post-transcriptional regulatory mechanisms are responsible for the observed gene-protein discrepancies.

To determine whether ErbB signaling drives the increased tumorigenesis observed in *Tpl2*^−/−^ mice, we evaluated the effects of EGFR or HER2 inhibition on tumor development and cSCC progression in our model. *Tpl2*^−/−^ mice developed 12-fold more papillomas and 4-fold more cSCCs than Tpl2^+/+^ animals. However, while substantial numbers of papillomas formed in *Tpl2*^−/−^ mice, only four cSCCs developed in untreated *Tpl2*^−/−^ mice compared to one in *Tpl2*^+/+^ mice and one in drug-treated *Tpl2*^−/−^ mice. These limited cSCC counts constrained our molecular characterization of the malignant lesions. Future investigations will necessitate larger animal cohorts and potentially longer observation periods to obtain adequate cSCC numbers for comprehensive analysis of therapeutic effects on malignant progression.

Treatment with the EGFR inhibitor Gefitinib or EGFR/HER2 inhibitor Lapatinib resulted in a decrease in the number of papillomas by 88% and 50%, respectively, and restored the number of cSCCs back to *Tpl2*^+/+^ levels. The superior efficacy of Gefitinib compared to Lapatinib suggests that EGFR signaling may be the predominant driver of tumorigenesis in this model, consistent with our observation that p-EGFR was the most strongly upregulated phospho-receptor in Tpl2^−/−^ papillomas. Our research extends prior studies showing clinical efficacy of gefitinib and lapatinib in cSCC treatment and provides mechanistic evidence supporting EGFR as a primary therapeutic target in *Tpl2*-deficient tumors [33,34].

## 5. Conclusions

In summary, we propose that compensatory upregulation of ErbB signaling plays a significant role in driving cSCC development when *Tpl2* is absent. These findings suggest that ErbB inhibition warrants further investigation as a potentially promising and targeted therapeutic intervention for this specific subset of cSCC tumors characterized by disruptions in MAPK signaling.

## Figures and Tables

**Table 1:** qPCR Primer Sequences for mRNA Targets (mus musculus).

Gene Name	EGFR	HER2	HER3	GAPDH
Forward Sequence (5′ to 3′)	GCCATCTGGGCCAAAGATACC	ACATGCTTCGCCACCTCTAC	CGGTTCCGGAGGGGATTATG	CATCACTGCCACCCAGAAGACTG
Reverse Sequence (5′ to 3′)	GTCTTCGCATGAATAGGCC	AGCTGAGTCCCTCTCACGAT	TGCCAGTAATCGGGGTTGTC	ATGCCAGTGAGCTTCCCGTTCAG

**Table 2:** microRNA sequences (mus musculus) used for quantification of miR21 and miR205.

miR	miR21 (mmu-miR-21a-3p)	miR205 (mmu-miR-205-3p)	miR7 (mmu-miR-7b-3p)	miR125 (mmu-miR-125b-5p)
Sequence (Mature)	CAACACAGUCGAUGGGCUGUC	GAUUUCAGUGGAGUGAAGCUCA	UGGAAGACUUGUGAUUUUGUUGUU	UCCCUGAGACCCUAACUUGUGA

**Table 3:** Histological Analysis of Tumor Specimens from Chemical Carcinogenesis Study.

	Papillomas	Squamous Cell Carcinomas	Other	Total
*Tpl2*^+/+^ normal diet	3	1	0	4
*Tpl2*^−/−^ normal diet	46	4	0	50
*Tpl2*^−/−^ Gefitinib diet	4	1	1 (Atypical verrucous keratosis)	6
*Tpl2*^−/−^ Lapatinib diet	24	1	0	25

## Data Availability

The original data presented in the study are available in supplementary data and the OSF repository. DOI 10.17605/OSF.IO/CBYQD.

## Supplementary Materials

The following supporting information can be downloaded at: https://www.mdpi.com/article/10.3390/cancers17203362/s1, Figure S1: Full sized Western blots; Figure S2: mTOR blot; Figure S3: GAB1 and Ras blot; Table S1: IHC quantification.
