# Supplementary material for: Increased EGFR/HER2 Pathway Activation Contributes to Skin Tumorigenesis in Tpl2−/− Mice
Source: Cancers (Basel). 2025 Oct 18;17(20):3362. doi: 10.3390/cancers17203362 (PMC12563468; doi:10.3390/cancers17203362)
Supplement: Supplementary file 1 [file cancers-17-03362-s001.zip › cancers-3883005-supplementary.pdf]

Figure S1: Full Sized Western blots

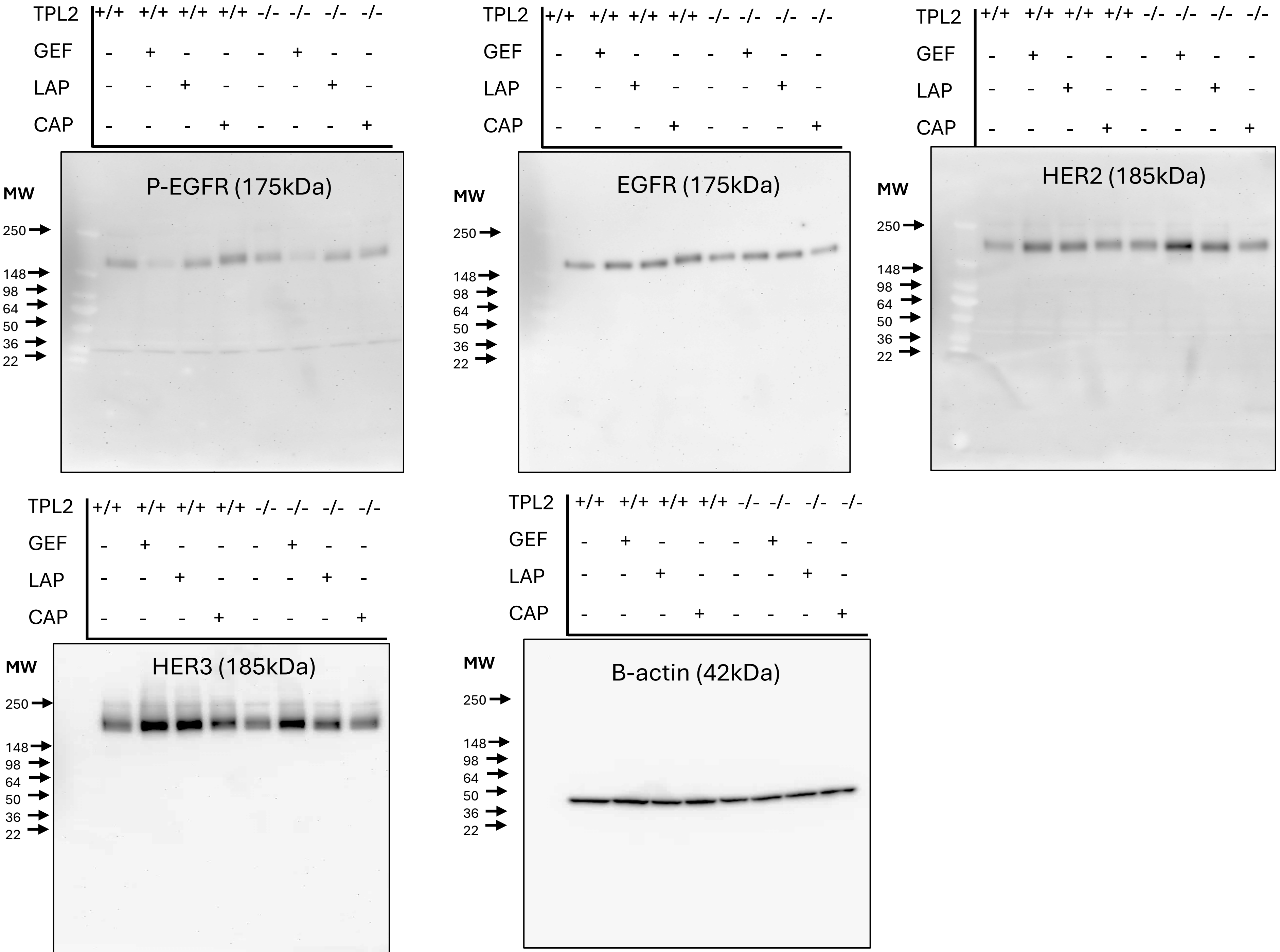

Figure S2: MTOR Western

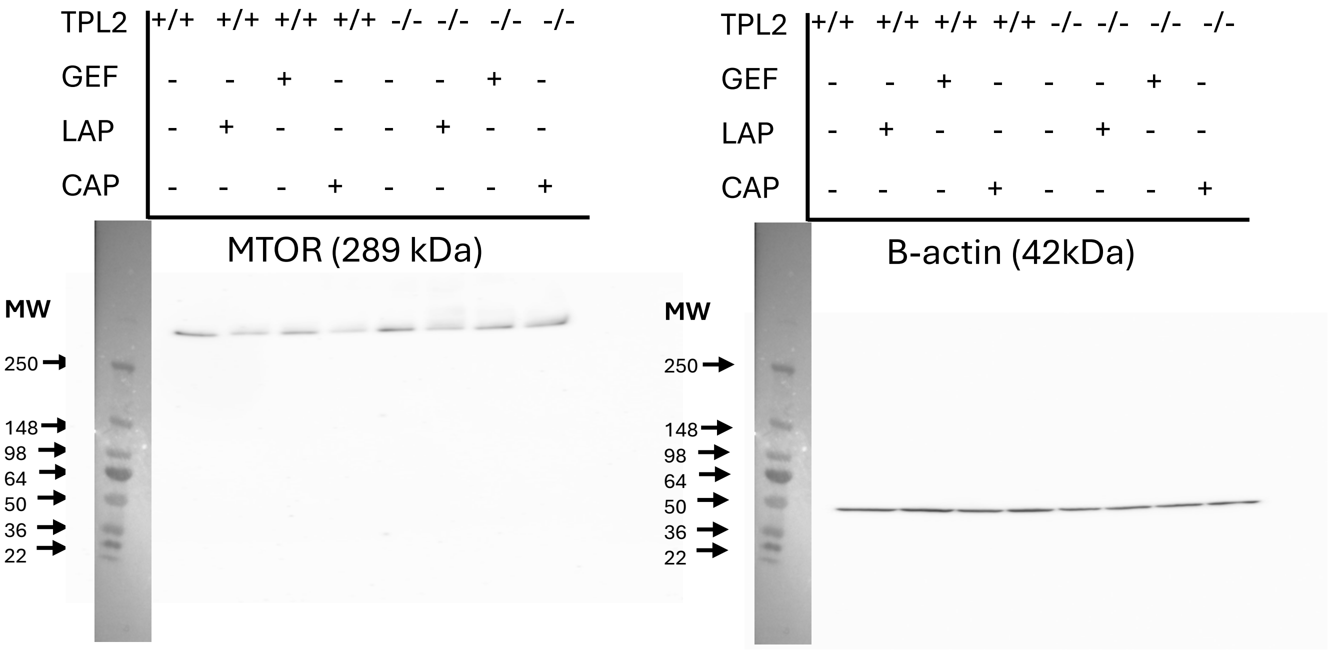

Figure S3: Gab1 and Ras Western

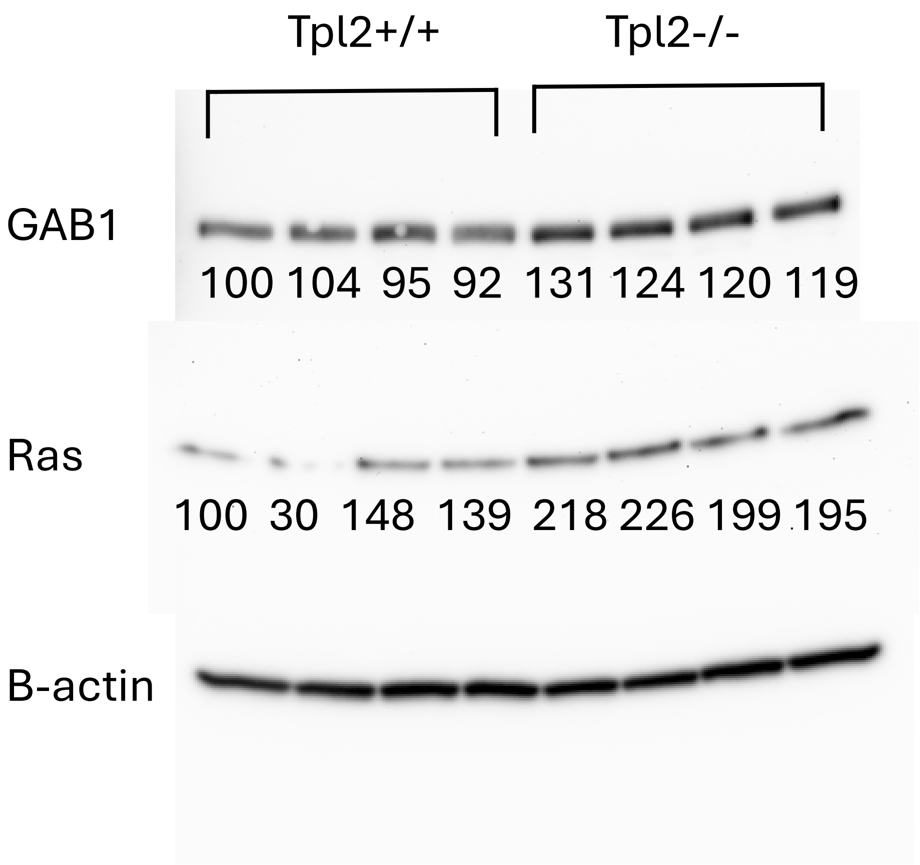

Table S1: IHC quantification

Condition	Total area of stained puncta (um^2) (from insets)	Raw Area from ImageJ
WT EGFR Skin	614.2408	19.438
WT EGFR Papilloma	7640.7852	241.797
WT EGFR SCC	6537.25	206.875
KO EGFR Skin	376.8932	11.927
KO EGFR Papilloma	15508.174	490.765
KO EGFR SCC	4793.5936	151.696
WT HER2 Skin	135.7852	4.297
WT HER2 Papilloma	8716.1964	275.829
WT HER2 SCC	3232.2376	102.286
KO HER2 Skin	92.5248	2.928
KO HER2 Papilloma	14766.4588	467.293
KO HER2 SCC	3495.434	110.615
WT HER3 Skin	274.5408	8.688
WT HER3 Papilloma	7405.7444	234.359
WT HER3 SCC	5004.3024	158.364
KO HER3 Skin	547.47	17.325
KO HER3 Papilloma	12035.1444	380.859
KO HER3 SCC	190.5164	6.029
WT pEGFR Skin	339.3524	10.739
WT pEGFR Papilloma	6662.3544	210.834
WT pEGFR SCC	6084.8644	192.559
KO pEGFR Skin	119.0056	3.766
KO pEGFR Papilloma	14208.3712	449.632
KO pEGFR SCC	636.7084	20.149
WT pHER2 Skin	57.3224	1.814
WT pHER2 Papilloma	4143.2024	131.114
WT pHER2 SCC	3754.396	118.81
KO pHER2 Skin	40.4796	1.281
KO pHER2 Papilloma	1621.4908	51.313
KO pHER2 SCC	1834.854	58.065
WT pHER3 Skin	821.916	26.01
WT pHER3 Papilloma	6410.9132	202.877
WT pHER3 SCC	2096.66	66.35
KO pHER3 Skin	465.6576	14.736
KO pHER3 Papilloma	4213.8284	133.349
KO pHER3 SCC	1425.7604	45.119
